# The Three Models of Emotional Intelligence and Performance in a Hot and Cool go/no-go Task in Undergraduate Students

**DOI:** 10.3389/fnbeh.2017.00033

**Published:** 2017-02-22

**Authors:** María J. Gutiérrez-Cobo, Rosario Cabello, Pablo Fernández-Berrocal

**Affiliations:** ^1^Department of Basic Psychology, Faculty of Psychology, University of MálagaMálaga, Spain; ^2^Department of Developmental and Educational Psychology, University of GranadaGranada, Spain

**Keywords:** emotional intelligence, cognitive control, go/no-go tasks, hot tasks, cool tasks

## Abstract

Emotional intelligence (EI), or the ability to perceive, use, understand and regulate emotions, appears to be helpful in the performance of “hot” (i.e., emotionally laden) cognitive tasks when using performance-based ability models, but not when using self-report EI models. The aim of this study is to analyze the relationship between EI (as measured through a performance-based ability test, a self-report mixed test and a self-report ability test) and cognitive control ability during the performance of hot and “cool” (i.e., non-emotionally laden) “go/no-go” tasks. An experimental design was used for this study in which 187 undergraduate students (25% men) with a mean age of 21.93 years (standard deviation [SD] = 3.8) completed the three EI tests of interest (Mayer-Salovey-Caruso Emotional Intelligence Test [MSCEIT], Trait Meta-Mood Scale [TMMS] and Emotional Quotient Inventory–Short Form [EQi:S]) as well as go/no-go tasks using faces and geometric figures as stimuli. The results provide evidence for negative associations between the “managing” branch of EI measured through the performance-based ability test of EI and the cognitive control index of the hot go/no-go task, although similar evidence was not found when using the cool task. Further, the present study failed to observe consistent results when using the self-report EI instruments. These findings are discussed in terms of both the validity and implications of the various EI models.

## Introduction

For centuries, emotion and cognition were understood as separate concepts. Whilst emotion was traditionally regarded as a primitive mechanism, cognition, on the other hand, was viewed as the more complex aspect of the human psyche (Ekman and Davidson, 1994). Today, however, the notion of an interactive and bidirectional relationship between both constructs has gained wide acceptance. Neuroscientists have revealed complex interactions between the two processes, demonstrating a high level of interdependence between them (Phelps et al., 2014). Thus, emotions and cognitive processes such as attention, decision making and memory (among others) appear to be related (Lerner et al., 2015). For instance, a negative emotional state will promote more systematic, detailed and careful processing of information, while a positive emotional state will lead to a less systematic (but more creative and spontaneous) processing style (Bless et al., 1990; Schwarz and Bless, 1991).

One construct that attempts to connect the concepts of emotion and cognition is what is known as “emotional intelligence” (EI). Mayer and Salovey (1997; p. 10), have provided perhaps the most relevant approach to this concept, and define it as

… the ability to perceive accurately, appraise and express emotion; the ability to access and/or generate feelings when they facilitate thought; the ability to understand emotion and emotional knowledge; and the ability to regulate emotions to promote emotional and intellectual growth.

Since its introduction by Salovey and Mayer (1990), EI has been the subject of much empirical work. However, this growing interest has both advantages and disadvantages. One advantage is the enormous resources that investigators have invested in studying the concept, which has led to vast amounts of models, instruments and investigations. However, this research has not always been conducted in a systematic and rigorous scientific manner, which has hindered progress in the conceptualization of the construct (Mayer et al., 2008b). In an attempt to organize the EI literature, Joseph and Newman (2010) proposed three models, which can be distinguished according to the type of measuring instruments that have been employed.

In the first, the *performance-based ability model*, EI is viewed as a form of intelligence that is based on emotional aptitudes, and is regarded as a mental ability that involves reasoning about our emotions, which is focused on hot information processing (Mayer et al., 2016). Within this framework, EI is evaluated by solving emotional problems through performance tests that include a set of correct and incorrect responses. The most representative instrument of this model is the “Mayer-Salovey-Caruso Emotional Intelligence Test” (MSCEIT; Mayer et al., 2002). The second model is the *self-report ability model*, which, like the performance-based ability model, views EI as a combination of emotional aptitudes; in this case, however, self-report instruments are used, where participants must estimate their own EI in a subjective manner (Fernández-Berrocal and Extremera, 2008). Thus, there are no correct and incorrect responses in the self-report ability model, the “Trait Meta-Mood Scale” (TMMS; Salovey et al., 1995) being a widely used instrument for this approach. Finally, the *self-report mixed model* does not consider EI to be a form of intelligence but instead views it as a broad concept that includes (among others) motivations, interpersonal and intrapersonal abilities, empathy, personality factors and well-being (Mayer et al., 2008a). Again, this model employs self-report instruments that evaluate the subjective perception of the participants; the “Bar-On Emotional Quotient Inventory” (EQi)—named after its creator, Bar-On (2004)—is a commonly employed test for this model. However, some researchers have questioned the self-report mixed model, arguing that it is unhelpful to conceptualize EI in terms of old (and already studied) concepts, since such an approach fails to provide any new information (Locke, 2005). In addition, although the three models essentially assess the same construct, any correlations between them appear to be weak (Goldenberg et al., 2006; Webb et al., 2013; Cabello and Fernández-Berrocal, 2015a).

Despite the differences between these models, their use has yielded a large number of EI-related outcomes. Thus, researchers have linked higher EI scores to better mental and physical health (Schutte et al., 2007; Martins et al., 2010; Zeidner et al., 2012), well-being and happiness (Cabello and Fernández-Berrocal, 2015b; Sánchez-Álvarez et al., 2015), job performance (Côté, 2014), prosocial behavior (Mavroveli et al., 2009), less aggressive behavior (García-Sancho et al., 2014), and substance abuse (Kun and Demetrovics, 2010). One relationship that researchers have not examined in great detail, however, is that between EI and cognitive processes. One question that arises in this context is whether individuals with higher EI will perform better on cognitive tasks.

In order to explore this relationship, Gutiérrez-Cobo et al. (2016) recently conducted a systematic review of the literature. The authors divided the 26 studies they found into two categories: EI instruments (performance-based ability test, self-report ability test and self-report mixed test) and cognitive processes (“hot” or emotionally laden vs. “cool” or non-emotionally laden). The authors found that performance-based ability EI (but not the self-report EI test) was positively correlated with efficiency in hot cognitive tasks, but not cool cognitive tasks, which suggests that higher EI could indicate cognitive efficiency, depending on the emotional content. However, Gutiérrez-Cobo et al. (2016) have pointed out that these results were inconclusive for several reasons. The first of these relates to the vast number of different EI instruments employed (a total of 13 scales) as well as the large variability in cognitive tasks (a total of 18 tasks) that were used. Second the studies included a range of cognitive processes (attention, memory, decision making, etc.) that could have been influenced by the EI construct in different ways. Finally, only a few studies have analyzed cool cognitive tasks, making it difficult to draw any firm conclusions.

On the basis of the limitations highlighted by Gutiérrez-Cobo et al. (2016), the aim of the present study was to evaluate the relationship between EI using the three models discussed above and a specific hot and cool cognitive capacity known as “cognitive control ability”. Cognitive control describes a heterogeneous construct that allows the representation and attainment of goal-directed behaviors as well as the detection and resolution of conflicts in information processing (Miller and Cohen, 2001). This ability is fundamental to people’s daily activities, and it allows for flexibility in their behavior. Deficits in cognitive control could, for instance, lead to problems related to impulsiveness, drug abuse, or caffeine over-consumption (Aichert et al., 2012; Volkow et al., 2013; Holmes et al., 2016). In addition, EI has been shown to be a beneficial moderator during stressful situations as well as a protective factor for these risky behaviors, in which a lack of cognitive control plays a key role (Slaski and Cartwright, 2003; Casey et al., 2011; Schneider et al., 2013; Extremera and Rey, 2015; Limonero et al., 2015; Peña-Sarrionandia et al., 2015).

Cognitive control is not a unitary neurocognitive construct. It consists of two different processes: interference control and motor response inhibition. When discussing interference control, we refer to the ability to prevent attentional interference due to competition between relevant and irrelevant stimuli (Nigg, 2000). Motor response inhibition includes the ability to restrain a strong response or cancel an ongoing response (Schachar et al., 2007). Given this heterogeneity, a large number of tasks are used to measure cognitive control ability, including the Stroop task (Stroop, 1935), the stop-signal task (Logan et al., 1984), and the go/no-go task (Van der Meere et al., 1995). Whilst these tasks all inhibit the processing of an inadequate “prepotent” response, they involve quite different mechanisms. In particular, the latter two measures are part of the motor response inhibition construct, whilst the Stroop task measures interference control. In a typical Stroop task, participants must indicate the color of a given word in which the word and the color are incompatible—for instance, the word “red” written in blue ink. The subject’s reaction time (RT) will be longer when the color and word are incongruent than when they are not (for example, the word “red” written in red ink). Since the introduction of the original Stroop task, researchers have developed numerous versions, including those that utilize a large variety of stimuli. Few studies have evaluated the relationship between EI and Stroop tasks. For instance, Coffey et al. (2003), using a self-report ability model instrument, reported longer RTs for participants who exhibited higher levels of attention to feeling, whilst Martin and Thomas (2011) and Checa and Fernández-Berrocal (2015) both found a negative correlation between a performance-based ability model instrument and an RT index of the Stroop task.

Our first objective in the present study is to evaluate the relationship between EI and cognitive control abilities using a task that is more centered on the motor aspect of cognitive control, namely the go/no-go task. This procedure evaluates the ability to withhold a prepotent response, and to the best of our knowledge, no studies have yet used this paradigm to analyze the relationship between EI and cognitive control. We selected the go/no-go task given that it shows reliable and robust results (Casey et al., 1997). Our second aim is to assess whether or not this relationship depends on the emotional content of the task by using cool and hot go/no-go tasks (Schulz et al., 2007). Finally, we wanted to determine which EI model is the most predictive of this cognitive process by using three EI instruments: the performance-based ability model, the self-report ability model and the self-report mixed model. We hypothesize that individuals with a high level of development of the EI construct—measured via a performance-based ability model instrument but without self-report measures—will display higher cognitive control abilities in the emotional task. In other words, by using this performance instrument, we expect to find a lower false alarm (FA) rate in the hot go/no-go task.

These results are to be expected due to the nature of the EI performance instruments, which use objective measures that are the equivalent of the cognitive control task, and as such, participants cannot falsify their responses. Through self-reports, however, participants give their subjective opinion about the status of their EI and, therefore, such responses may be inaccurate. Our current hypothesis is also based on the results of previous research (Gutiérrez-Cobo et al., 2016). In particular, neuroscience studies have demonstrated that the same frontal regions of the brain are involved in both cognitive control and the processing related to the performance-based ability model (Miller, 2000; Jausovec et al., 2001; Ridderinkhof et al., 2004; Rushworth et al., 2004; Krueger et al., 2009; Tang et al., 2016). Finally, due to the emotional nature of the cognitive task (both hot and cool), we expect that EI will be related to the hot cognitive task but not to the cool task. As Gutiérrez-Cobo et al. (2016) have found, it appears that the beneficial effect of EI depends on emotional content. This result is also compatible with the various definitions of the EI concept, which is understood to be a form of intelligence that helps individuals to cope with both their own emotions and those of others in an adaptive manner (Mayer and Salovey, 1997).

## Materials and Methods

### Participants and Procedure

The sample consisted of 199 psychology undergraduates from the University of Málaga, Spain (25% men), ranging in age from 19 to 48 years (*M* = 21.87, standard deviation [SD] = 3.82). Due to the cut-off employed, the final sample was composed of 187 participants. Inclusion criteria were the absence of any motor or visual disability and mental retardation. They took part in the experiment in exchange for course credits. The study was carried out in accordance with the Declaration of Helsinki and ethical guidelines of the American Psychological Association, and all participants provided written informed consent. The Research Ethics Committee of the University of Málaga approved the study protocol as part of the projects SEJ-07325 and PSI2012-37490. The experiment was carried out in three different stages. First, the participants completed the MSCEIT in a collective classroom environment. Second, they completed the TMMS and Emotional Quotient Inventory–Short Form (EQi:S) online (from the final sample, seven and nine participants did not complete the TMMS and EQi-S, respectively). The two types of administration of the EI instruments did not affect their reliability (see Table 1). Finally, the participants performed the two cognitive tasks in a quiet room using one of ten semi-isolated computers. Both cognitive tasks were programmed using the software E-prime 2.0. Each participant was assigned a number in order to maintain anonymity.

**Table 1:** **Descriptive statistics of the emotional intelligence (EI) instruments**.

	Mean	Min.	Max.	SD	Median	Asymmetry	Kurtosis	**α**
**MSCEIT**
Total	108.98	86.49	124.38	8.21	110.63	−0.69	−0.02	0.82
Perceiving emotions	108.76	56.33	122.29	10.89	111.33	−1.80	5.01	0.78
Facilitating emotions	101.94	65.71	119.50	9.72	103.50	−1.06	1.50	0.72
Understanding emotions	108.44	69.73	127.00	9.79	110.56	−1.24	1.79	0.76
Managing emotions	109.33	71.91	124.20	10.81	111.64	−1.04	0.96	0.78
**TMMS**
Attention to feeling	3.52	1.00	5.00	0.54	3.63	−0.57	0.22	0.90
Clarity of feeling	3.41	1.00	5.00	0.74	3.50	−0.33	0.12	0.85
Mood repair	3.45	1.50	5.00	0.71	3.50	−0.30	0.03	0.85
**EQi:S**
Interpersonal	4.22	2.29	5.00	0.44	4.29	−0.63	1.46	0.78
Adaptability	3.82	1.20	5.00	0.67	3.80	−0.52	0.74	0.82
Stress management	3.32	1.13	4.88	0.85	3.38	−0.26	−0.51	0.87
Intrapersonal	3.68	1.50	5.00	0.69	3.75	−0.42	−0.02	0.81

### EI Instruments

*Mayer-Salovey-Caruso Emotional Intelligence Test* (MSCEIT v. 2.0; Mayer et al., 2002; Extremera and Fernández-Berrocal, 2009). For the performance-based ability model, we employed the Spanish translation of the MSCEIT, which shows psychometric properties similar to the original English-language instrument (Extremera et al., 2006). This test has been validated for adults aged 17 and above. The MSCEIT is a 141-item test that measures the four branches of the EI definitions proposed by Mayer and Salovey (1997): *perceiving, facilitating, understanding* and *managing* emotions. This instrument uses two tasks to measure each of the four branches of EI, comprising a total of eight tasks. An example of an item related to the facilitating branch is “What mood (s) might be helpful to feel when meeting in-laws for the very first time?” Here, participants have to choose, on a scale from 1 (not useful) to 5 (useful), how different emotions such as tension, surprise and joy would favor this situation. The instrument provides separate scores for each branch as well as an overall score for total EI. Scores can be calculated based on expert or consensus norms, which tend to correlate strongly with each other (*r* > 0.90; Mayer et al., 2003). Scores computed by the test publishers are standardized (*M* = 100, SD = 15); the reliability of the two halves is 0.93, based on the consensus criterion. The test-retest reliability of the global MSCEIT is 0.86 after 3 weeks (Brackett and Mayer, 2003). In the present study, we used consensus norms to calculate the scores for total EI ability as well as for each of the four branches. For the scales that were used in the present study, internal consistency ranged from 0.72 to 0.82 (perceiving = 0.78, facilitating = 0.72, understanding = 0.76, managing = 0.78 and total EI = 0.82). This consistency was measured as the Cronbach’s alpha (*α*) in the case of total EI score and as two-halves consistency in the case of the remaining scores.

*Trait Meta-Mood Scale* (TMMS; Salovey et al., 1995; Fernández-Berrocal et al., 2004). For the self-report ability model, we employed the Spanish translation of the TMMS (Fernández-Berrocal et al., 2004). This is a 24-item test that has been validated in a university student population, and measures individuals’ beliefs on three dimensions: *attention, clarity* and *repair* (Fernández-Berrocal and Extremera, 2008). An item example is “I don’t pay too much attention to my feelings” and participants have to respond on a Likert-scale from 1 (strongly disagree) to 5 (strongly agree). In our study, we found good reliability for the three dimensions: a Cronbach’s *α* of 0.90 for attention and clarity and 0.85 for repair.

*Emotional Quotient Inventory–Short Form* (EQi:S; Parker et al., 2011; López-Zafra et al., 2014). For the self-report mixed model, we employed the Spanish translation of the EQi:S (EQi:C; López-Zafra et al., 2014). This test has also been validated in a university student population. The EQi:S is a 28-item test that measures four dimensions of traits and self-concepts: *intrapersonal, interpersonal, adaptability* and *stress management*. Participants respond using a five-point Likert-type scale from 1 (never) to 5 (always) to statements such as “I like to help people”. The instrument provides a score for each dimension. The reliability for the four scores ranged from moderate to high: intrapersonal (*α* = 0.73), interpersonal (*α* = 0.78), adaptability (*α* = 0.70) and stress management (*α* = 0.75). In our study, we found an acceptable level of reliability for the interpersonal scale (*α* = 0.70) and a good level of reliability for adaptability (*α* = 0.82), stress management (*α* = 0.87) and intrapersonal scales (*α* = 0.81).

### Go/no-go Tasks

#### Hot go/no-go Task

**Figure 1:**
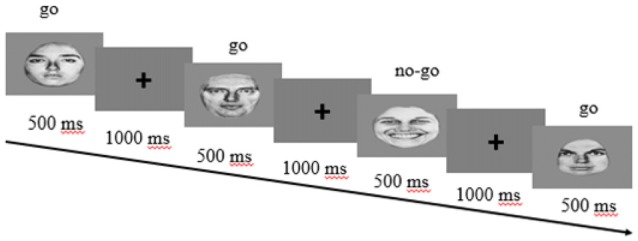
**Hot go/no-go task design**.

This task was adapted from the work of Tottenham et al. (2011). The set of stimuli was composed of gray scale images of adult females and males with five different expressions: angry, fearful, happy, sad and neutral (Ekman and Friesen, 1976). In this task (Figure 1), the participants pressed a button when a “go” stimulus (one of the five facial expressions) appeared on the screen, and they had to contain their responses when a “no-go” stimulus (a different facial expression) was presented. During each block, the participants were informed that a certain facial expression (e.g., an angry face) was the go stimulus. Participants conducted this task across a total of eight counter-balanced blocks: for four of the blocks, the go stimulus was always a given emotional expression (e.g., happy), while the no-go stimulus was a neutral expression. For the remaining four blocks, this pattern was reversed: the go stimulus was always a neutral face, while the no-go stimulus was a specific emotional expression (e.g., a fearful face). In addition, each block consisted of 30 trials in which the go stimuli occurred more frequently (70%) than the no-go stimuli (30%), which helped to create a tendency to respond to the go stimuli. During each trial, the stimuli appeared for 500 ms, with 1000 ms between trials during which participants could respond. Finally, before the task began, the participants completed a practice phase to familiarize themselves with the task.

#### Cool go/no-go Task

**Figure 2:**
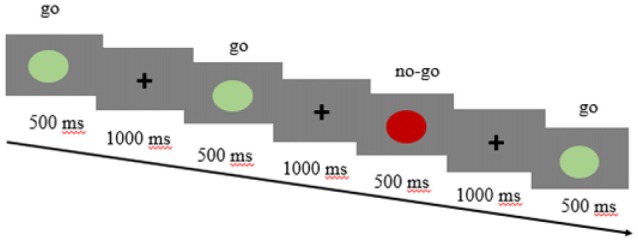
**Cool go/no-go task design**.

This task was performed in the same way as the hot go/no-go task but with neutral stimuli (Figure 2). The set of stimuli was composed of green and red circles. Only one block of 120 trials was conducted, and again, the go stimuli appeared more frequently (70%) than the no-go stimuli (30%).

### Data Analyses

All statistical analyses were conducted using SPSS 22.0 (IBM, Armonk, NY, USA). Those participants with total MSCEIT scores below 85 were removed from the analyses given that it indicates a misunderstanding of the task or an emotional deficit (Extremera et al., 2006). Preliminary analyses were carried to compute the EI descriptive statistics and the correlations among EI instruments (see “Emotional Intelligence” Section). In order to analyze the cool and hot cognitive tasks, we calculated RT, FA and d-prime indices. Participants with a total number of errors that were more than 2 SDs from the mean on each cognitive task were removed from the study (Luque et al., 2016). RT was calculated only for correct trials and trials with no more than ±3 SDs from the mean of each participant (Tottenham et al., 2011) in our sample. FA rates served as the measure of cognitive control; they were calculated as the percentage of responses that were given on the no-go trials. Finally, the d-prime measure provided a discrimination index and, following Tottenham et al. (2011), was obtained by subtracting the z-transformed FA rate from the z-transformed hit rate. We first conducted a within-subject ANOVA using emotion (angry, fearful, happy, sad) and stimulus type (emotion as “go” or “no-go”) for the RT, FA and d-prime indices (without including EI) to fully capture the effect of each condition (see “Hot go/no-go Task” Section) as well as *post hoc t*-test comparisons between emotions and stimulus type applying Bonferroni corrections, setting statistical significance at *p* < 0.006 (0.05/8). We then made bivariate correlations between the three EI variables and each of the hot go/no-go indices (see “Correlations” Section). In addition, in order to clarify the various correlations found, multiple regression analyses were used to test if the dimensions of the MSCEIT, TMMS and EQi-S significantly predicted participants’ total index of RT, FA and d-prime (see “Multiple Regressions” Section). Afterwards, bivariate correlations were conducted between the EI variables and the cool go/no-go RT, FA and d-prime indices (see “Cool go/no-go Task and EI” Section). Finally (see “Cool and Hot go/no-go Task” Section), in order to find any similarities between tasks, bivariate correlations and *t*-test comparisons were carried out between the cool and hot go/no-go cognitive task indices (total errors, FA, RT and d-prime).

## Results

### Emotional Intelligence

We first conducted descriptive analyses of the three EI instruments (Table 1). Correlations between the three EI instruments were also examined (Table 2), and these analyses revealed a positive and significant correlation between the management branch of the MSCEIT with the EQi-S intrapersonal scale (*r* = 0.15, *p* = 0.048) as well as the clarity (*r* = 0.16, *p* = 0.04) and repair (*r* = 0.19, *p* = 0.01) TMMS scales. The attention scale of the TMMS correlated positively with the interpersonal (*r* = 0.42, *p* < 0.01) and adaptability (*r* = 0.25, *p* = 0.002) scales of the EQi-S but negatively with the stress management scale (*r* = −0.20, *p* = 0.009). In addition, the TMMS clarity scale correlated positively with the EQi-S interpersonal (*r* = 0.24, *p* = 0.001), adaptability (*r* = 0.35, *p* < 0.01), stress management (*r* = 0.26, *p* < 0.01), and intrapersonal (*r* = 0.59, *p* < 0.01) scales. Finally, the repair scale of the TMMS correlated positively with the interpersonal (*r* = 0.32, *p* < 0.01), adaptability (*r* = 0.35, *p* < 0.01), and intrapersonal (*r* = 0.38, *p* < 0.01) scales of the EQi-S.

**Table 2:** **Pearson correlations among the Mayer-Salovey-Caruso emotional intelligence test (MSCEIT), trait meta-mood scale (TMMS), and emotional quotient inventory–short form (EQi-S) sub-scales**.

	1	2	3	4	5	6	7	8	9	10	11
**MSCEIT**
1. Total
2. Perceiving emotions	0.72**
3. Facilitating emotions	0.70**	0.42**
4. Understanding emotions	0.60**	0.13	0.27**
5. Managing emotions	0.49**	0.03	0.16*	0.23**
**TMMS**
6. Attention to feeling	0.01	−0.09	0.02	0.02	0.12
7. Clarity of feeling	0.03	−0.10	0.04	0.05	0.16*	0.24**
8. Mood repair	0.12	0.02	0.11	0.02	0.19*	0.02	0.46**
**EQi:S**
9. Interpersonal	0.11	0.08	0.11	−0.01	0.12	0.42**	0.24**	0.32**
10. Adaptability	0.05	−0.02	0.00	0.07	0.10	0.23**	0.35**	0.35**	0.22**
11. Stress management	0.11	0.04	0.11	0.06	0.09	−0.20**	0.26**	0.12	−0.06	0.20**
12. Intrapersonal	0.10	0.01	0.12	0.03	0.15*	−0.04	0.59**	0.38**	0.24	0.29**	0.38**

*p = 0.005. **p = 0.001. 1 = MSCEIT total; 2 = Perceiving emotions MSCEIT; 3 = Facilitating emotions MSCEIT; 4 = Understanding emotions MSCEIT; 5 = Managing emotions MSCEIT; 6 = Attention to feeling TMMS; 7 = Clarity of feeling TMMS; 8 = Mood repair TMMS; 9 = Interpersonal EQi:S; 10 = Adaptability EQi:S; 11 = Stress management EQi:S; 12 = Intrapersonal EQi:S.

### Hot go/no-go Task

**Figure 3:**
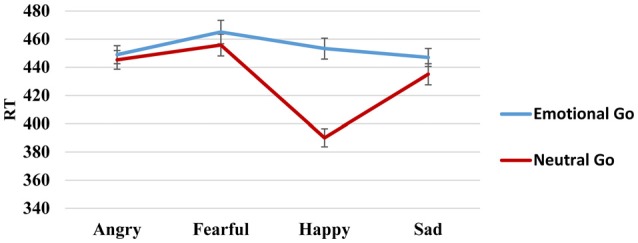
**Reaction time (RT) go/no-go task**.

A within-subject ANOVA was then conducted using the factors of emotion (angry, fearful, happy, sad) and stimulus type (emotion as “go” or “no-go”) for the RT, FA and d-prime variables. For RT (Figure 3), the ANOVA yielded a significant main effect of emotion (*F*_(3,531)_ = 33.66, *p* < 0.01, $\eta_{\text{p}}^{2}$ = 0.16), a main effect of stimulus type (*F*_(1,177)_ = 40.37, *p* < 0.01, $\eta_{\text{p}}^{2}$ = 0.19), and a significant interaction between emotion and stimulus type (*F*_(3,531)_= 24.84, *p* < 0.01, $\eta_{\text{p}}^{2}$ = 0.12). Using Bonferroni correction, RTs were fastest for happy stimuli (mean [SD]: 421.35 [6.49]; *p* (relative to angry) < 0.01; *p* (relative to fearful) < 0.01; *p* (relative to sad) < 0.01), followed by sad (mean [SD]: 440.25 [6.53]; *p* (relative to angry) = 0.71; *p* (relative to fearful) < 0.01) and angry (mean [SD]: 446.06 [6.15]; *p* (relative to fearful) = 0.01) faces; finally, fearful faces showed the slowest RTs (mean [SD]: 458.38 [7.33]). In addition, the participants were faster in responding to the go stimuli when the stimuli were neutral faces (mean [SD]: 430.80 [6.47]) than when they were emotional expressions (mean [SD]: 452.22 [6.43]; *p* < 0.01). In order to analyze the interaction, we carried out *post hoc* *t-tests* for each emotion to search for differences due to stimulus type. These analyses showed that RTs were longest when sad (*t*_(185)_ = 2.09, *p* = 0.04) and happy (*t*_(182)_ = 10.67, *p* < 0.01) faces were the go stimuli. No differences were found for angry (*t*_(185)_ = 0.68, *p* = 0.50) and fearful (*t*_(183)_ = 1.34, *p* = 0.18) faces.

**Figure 4:**
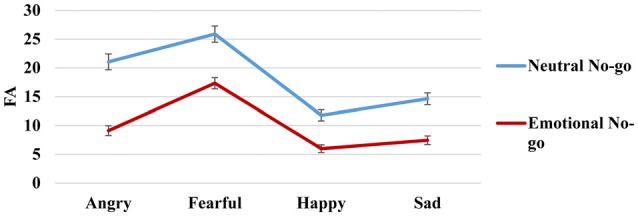
**False alarm (FA) go/no-go task**.

For FA rates (Figure 4), the ANOVA again showed a significant main effect of emotion (*F*_(3,558)_ = 67.14, *p* < 0.01, $\eta_{\text{p}}^{2}$ = 0.27) and stimulus type (*F*_(1,186)_ = 147.37, *p* < 0.01, $\eta_{\text{p}}^{2}$ = 0.44) as well as a significant emotion × stimulus type interaction (*F*_(3,558)_ = 3.89, *p* = 0.006, $\eta_{\text{p}}^{2}$ = 0.02). Using Bonferroni correction, FA scores were also higher for fearful stimuli (mean [SD]: 21.50 [0.99]; *p* (relative to angry) < 0.01; *p* (relative to happy) < 0.1; *p* (relative to sad) < 0.01), followed by angry (mean [SD]: 14.81 [0.86]; *p* (relative to happy) < 0.01; *p* (relative to sad) < 0.01) and sad (mean [SD]: 10.96 [0.70]; *p* (relative to happy) = 0.04) stimuli; happy stimuli showed the lowest FA rates (mean [SD]: 8.61 [0.60]). Moreover, the participants exhibited higher FAs for neutral no-go stimuli (mean [SD]: 17.97 [0.72]) than for emotional no-go stimuli (mean [SD]: 9.97 [0.52]; *p* < 0.01). In order to further explore the source of the interaction between emotion and stimulus type, we carried out *post hoc t*-tests for each emotion to analyze differences between stimulus types. These analyses revealed that FA rates were always higher when an emotional face was the go stimulus (angry: *t*_(186)_ = 7.45, *p* < 0.01; fearful: *t*_(186)_ = 5.70, *p* < 0.01; happy: *t*_(186)_ = 5.16, *p* < 0.01; sad: *t*_(186)_ = 6.12, *p* < 0.01).

**Figure 5:**
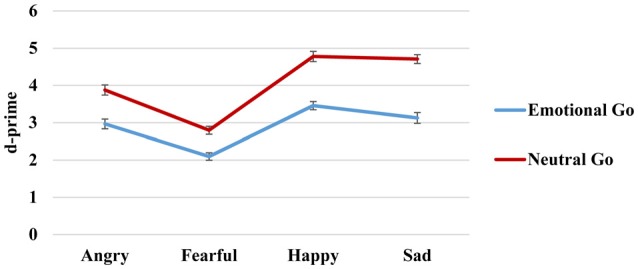
**D-prime hot go/no-go task**.

For d-prime scores (Figure 5), we again found a significant main effect of emotion (*F*_(3,558)_ = 83.60, *p* < 0.01, $\eta_{\text{p}}^{2}$ = 0.31) and stimulus type (*F*_(1,186)_ = 150.13, *p* < 0.01, $\eta_{\text{p}}^{2}$ = 0.45) as well as a significant emotion × stimulus type interaction (*F*_(3,558)_ = 6.49, *p* < 0.01, $\eta_{\text{p}}^{2}$ = 0.03). Using Bonferroni correction, the d-prime scores were again higher for happy (mean [SD]: 4.15 [0.09]; *p* (relative to angry) < 0.01; *p* (relative to fearful) < 0.01; *p* (relative to sad) = 0.44), sad (mean [SD]: 3.93 [0.10]; *p* (relative to angry) < 0.01; *p* (relative to fearful) < 0.01), and angry (mean [SD]: 3.41 [0.10]; *p* (relative to fearful) < 0.01) stimuli; fearful stimuli again showed the lowest d-prime scores (mean [SD]: 2.45 [0.08]). In addition, the participants showed better discrimination of neutral go stimuli (mean [SD]: 4.03 [0.07]) than emotional go stimuli (mean [SD]: 2.94 [0.08]; *p* < 0.01). *Post hoc t*-tests for each emotion on each stimulus type condition were conducted to further analyze the interaction. They showed that the d-prime scores were always higher when an emotional face was the no-go stimulus (angry: *t*_(186)_ = −4.82, *p* < 0.01; fearful: *t*_(186)_ = −4.84, *p* < 0.01; happy: *t*_(186)_ = −7.22, *p* < 0.01; sad: *t*_(186)_ = −9.09, *p* < 0.01).

### Hot go/no-go Tasks and EI

#### Correlations

##### MSCEIT

For RTs, we found significant and positive correlations between the managing branch and the total RT (*r* = 0.19, *p* = 0.001), the conditions in which the go stimulus was an emotional face (*r* = 0.18, *p* = 0.02) or a neutral face (*r* = 0.19, *p* = 0.01). When we focused on the type of emotion, we found that participants with higher scores for the managing branch showed longer RTs when the go stimuli were angry (*r* = 0.15, *p* = 0.04) and sad faces (*r* = 0.19, *p* = 0.01); this was also the case when the go stimulus was a neutral face and the no-go stimuli were angry (*r* = 0.17, *p* = 0.02), fearful (*r* = 0.17, *p* = 0.02), happy (*r* = 0.17, *p* = 0.02), or sad (*r* = 0.17, *p* = 0.02) faces.

For the FA, we found significant and negative correlations between the percentage of total FAs and the managing branch (*r* = −0.18, *p* = 0.02) and the total MSCEIT score (*r* = −0.15, *p* = 0.045). In addition, the participants with higher scores on the managing branch demonstrated lower FAs when the no-go stimuli were emotional (*r* = −0.15, *p* = 0.04) and neutral faces (*r* = −0.15, *p* = 0.04). When we focused on the kind of emotion that was presented, for those conditions in which the go stimuli were angry and happy faces, FA rates negatively and significantly correlated with the total scores (*r* = −0.19, *p* = 0.01; *r* = −0.15, *p* = 0.04, respectively) and the managing branch (*r* = −0.20, *p* = 0.01; *r* = −0.22, *p* = 0.002, respectively). We also found negative and significant correlations between the FA scores when the no-go stimulus was an angry face and the branch was managing (*r* = −0.14, *p* = 0.049); when the no-go stimulus was a happy face and the branches were understanding (*r* = −0.18, *p* = 0.01) and managing (*r* = −0.18, *p* = 0.02); and when the no-go stimulus was a sad face and the branch was perceiving (*r* = −0.15, *p* = 0.047).

For the d-prime, we found significant and positive correlations between total d-prime scores and the perceiving (*r* = 0.18, *p* = 0.02) and managing (*r* = 0.17, *p* = 0.02) branches and total MSCEIT scores (*r* = 0.20, *p* = 0.01). We also observed a significant and positive correlation between total EI scores and d-prime scores when the go stimulus was an emotional face (*r* = 0.17, *p* = 0.02). When we examined the type of emotion presented, when the go stimulus was an angry face, participants who scored higher on the managing branch tended to discriminate better than those who scored lower on this branch (*r* = −0.16, *p* = 0.03); the same outcome was obtained when the no-go stimulus was an angry face, but with the total score of the MSCEIT (*r* = −0.15, *p* = 0.04).

##### TMMS

For RTs, we found negative correlations approaching significance between the “repair” scale and the conditions in which the go stimulus was a neutral face (*r* = −0.14, *p* = 0.05). For the type of emotion, we found that participants with higher scores on the repair scale exhibited shorter RTs when the go stimulus was an angry face (*r* = −0.16, *p* = 0.03) and when the no-go stimulus was a sad face (*r* = −0.18, *p* = 0.02). For FA scores, we only achieved a significant and positive correlation between FAs when the go stimulus was a sad face and the scale was “attention” (*r* = 0.17, *p* = 0.02). For the d-prime, we again found only a significant and negative correlation between the d-prime score when the no-go stimulus was an angry face and the scale used was attention (*r* = −0.19, *p* = 0.01).

##### EQi-S

For RTs, we found a significant and negative correlation between the interpersonal scale and the conditions in which the go stimuli were emotional faces (*r* = −0.15, *p* = 0.04). When we focused on each emotion, we found that those participants who scored higher on the interpersonal scale had faster responses when the go stimuli were angry (*r* = −0.18, *p* = 0.02) and sad faces (*r* = −0.16, *p* = 0.04), while we found a positive correlation between the “stress management” scale and RTs when the go stimulus was a sad face (*r* = 0.16, *p* = 0.03). For the FA, we only found a significant correlation when focusing on particular emotions. Thus, these analyses revealed a negative correlation between the stress management scale and the conditions in which the go stimuli were happy (*r* = −0.15, *p* = 0.04) and sad faces (*r* = −0.19, *p* = 0.01) as well as when the no-go stimulus was a fearful face (*r* = −0.18, *p* = 0.02). For the d-prime, we again found only significant and positive correlations if we focused on the specific emotions. In particular, when the no-go stimulus was a fearful expression, participants with higher scores on the stress management (*r* = 0.19, *p* = 0.01) and intrapersonal (*r* = 0.17, *p* = 0.02) scales showed better discriminative performance than those with lower scores on those scales, as well as those with higher scores on the “adaptability” scale for the condition in which the no-go stimulus was a sad face (*r* = 0.19, *p* = 0.02).

#### Multiple Regressions

In order to clarify the various correlations found, multiple regression analyses were used to test if the dimensions of the MSCEIT, TMMS and EQi-S significantly predicted participants’ total index of RT, FA and d-prime. Prior to conducting this statistic analyses, relevant assumptions were tested. Specifically, assumption of multicollinearity was met as the tolerance and variance inflation factor (VIF) statistics were all within accepted limits. The assumption of singularity was also met as the EI variables do not reveal being highly correlated (see Table 2). Finally, the assumptions of independence measured with the Durbin-Watson statistic was also satisfied.

The results for the RT index indicated that there were two predictors: the management branch of the MSCEIT and the repair scale of the TMMS. These predictors explained 5% of the variance (adjusted *R*^2^ = 0.05, *F*_(2,174)_ = 5.52, *p* = 0.005). It was found that participants with higher scores on the managing branch showed longer RTs (*β* = 0.21, *p* = 0.006), while the opposite was found for the repair scale of the TMMS (*β* = −0.17, *p* = 0.02). The other EI index scores showed no significant association with RTs.

The results for the FA index indicated that the MSCEIT management branch was the only predictor. This predictor explained 2% of the variance (adjusted *R*^2^ = 0.02, *F*_(1,175)_ = 5.29, *p* = 0.02). In particular, higher scores on the managing branch significantly predicted less FA (*β* = −0.17, *p* = 0.02). No other indices predicted total FA rate.

Finally, we found two predictors for the d-prime index: the perceiving and management branches of the MSCEIT. These predictors explained 5% of the variance (adjusted *R*^2^ = 0.05, *F*_(2,174)_ = 5.83, *p* = 0.004). We found that higher scores on the perceiving branch significantly predicted higher total d-prime (*β* = 0.20, *p* = 0.008), as did the management branch (*β* = 0.15, *p* = 0.04). No other indices predicted total d-prime rate.

### Cool go/no-go Task and EI

Correlational analyses were also conducted for the three EI instruments using the RT, FA and d-prime variables in the cool go/no-go task. Significant correlations were found only between the “perceiving” branch of the MSCEIT and RTs (*r* = −0.17, *p* = 0.02). In this case—and contrary to what we found in the case of emotional stimuli—participants with higher EI scores took less time to complete each task.

### Cool and Hot go/no-go Task

**Figure 6:**
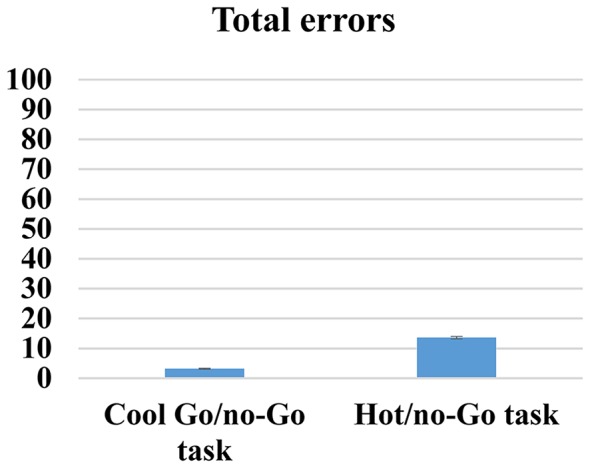
**Cool and hot go/no-go total errors**.

**Figure 7:**
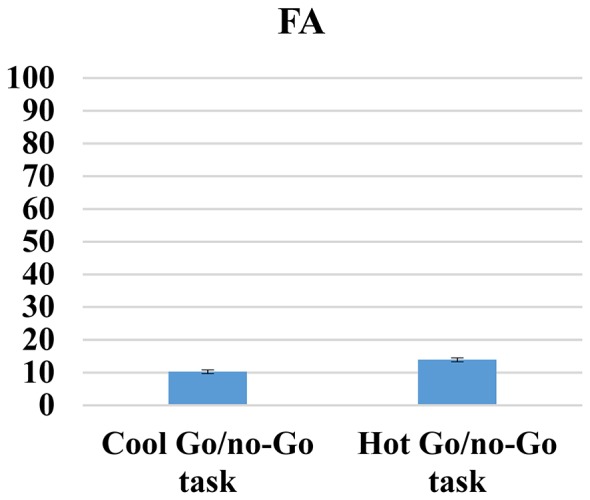
**Cool and hot go/no-go FAs**.

**Figure 8:**
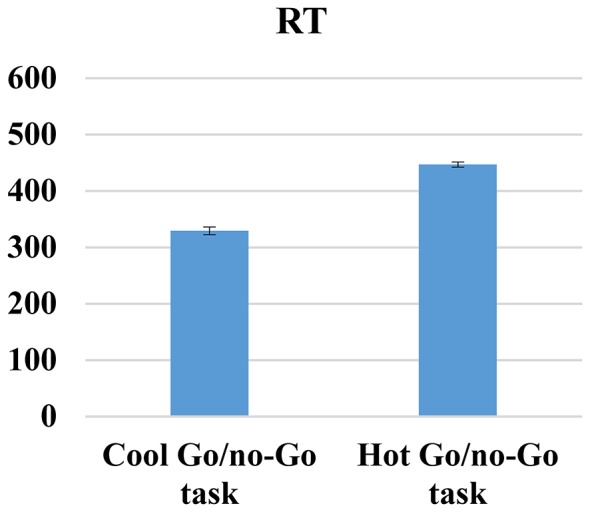
**Cool and hot go/no-go RT**.

**Figure 9:**
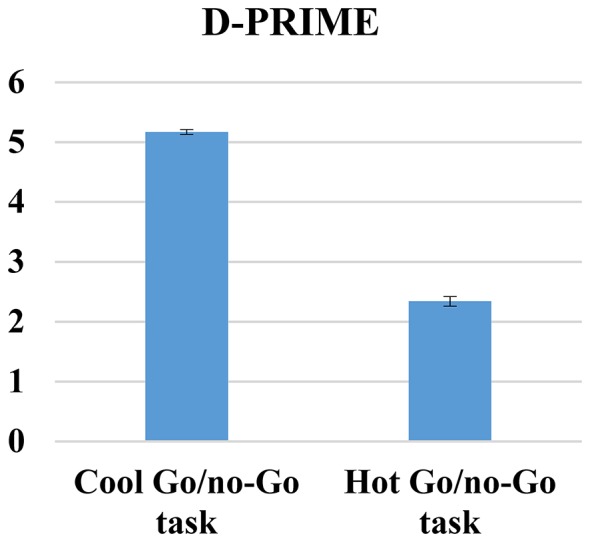
**Cool and hot go/no-go D-prime**.

First, given that both cool and hot go/no-go tasks are designed to measure the same cognitive processes, we correlated the total errors, RTs, FAs and d-prime variables between both tasks, and we only found positive and significant correlations between cool and hot go/no-go tasks for RT scores (*r* = 0.50, *p* < 0.01). No significant correlations were found for total errors (*r* = 08; *p* = 0.33), FA (*r* = 14; *p* = 0.07) and d-prime (*r* = 14; *p* = 0.08). We then conducted paired *t*-test comparisons to determine whether or not there were any differences in difficulty between the tasks; we found significant differences between all of the variables. Thus, participants made fewer total errors (*t*_(171)_ = 23.54, *p* < 0.01) and had fewer FAs (*t*_(171)_ = 4.75, *p* < 0.01); they spent less time (*t*_(171)_ = 19.52, *p* < 0.01); and they achieved higher d-prime scores (*t*_(171)_ = −33.44, *p* < 0.01) when responding to cool go/no-go tasks (total error mean [SD]: 3.18 [2.46]; FA mean [SD]: 10.26 [7.93]; RT mean [SD): 329.32 [60.51]; d-prime mean [SD]: 5.17 [1.06]) than to hot go/no-go tasks (total error mean [SD]: 13.62 [5.46]; FA mean [SD]: 13.92 [7.50]; RT mean [SD]: 446.68 [89.48]; d-prime mean [SD]: 2.34 [0.51]) These results are indicative of the higher level of difficulty of hot cognitive tasks compared with cold cognitive tasks, which is unsurprising (see Figures 6–9).

## Discussion

The present study analyzed the relationship between the three EI models and cognitive control abilities. In order to measure EI, we employed a performance-based ability model, a self-report ability model, and a self-report mixed model instrument. For assessing cognitive control, we utilized go/no-go tasks with emotional (hot) and non-emotional (cool) stimuli.

When we focused on the hot go/no-go task, we found a positive correlation between the managing branch of the MSCEIT and the RT index for the majority of the conditions (exceptions were the fearful go and happy go stimuli) and with the stress-management branch of the EQi-S for the sad go condition. In contrast, we found, for RT scores, a negative correlation between the repair dimension of the TMMS (angry go, sad no-go and overall neutral go) and the interpersonal dimension of the EQi-S (angry go, sad go and emotional go). With both self-report tasks, however, this correlation was only significant in three of the ten analyzed conditions, and was therefore not particularly consistent throughout the test blocks. In addition, multiple regression analyses support the correlational results for the managing and repair scales. These results show that higher-EI participants, as measured through the MSCEIT, took more time to respond to the go stimuli, thus suggesting that in these participants there is deeper processing of the facial expressions compared with their lower-EI counterparts. Given that the aim of the task was to focus not on the *speed* of the response but rather on its accuracy, taking longer to process each face would appear to be an adaptive strategy that allows the individual more time to choose the correct response. With the repair scale of the TMMS, in contrast, participants who perceived themselves as having higher EI had shorter RTs than those who perceived themselves as having lower EI. This result could be taken to imply that perceptions about one’s own EI ability are related to a less reflexive strategy in the cognitive task.

For our cognitive control index, with respect to the predictions of our hypothesis, we found that higher EI (primarily for the managing branch), measured with the MSCEIT, was related to lower FA rates (exceptions included fearful and sad go and fearful no-go stimuli). In addition, higher FA rates were related to lower scores in the stress-management scale of the EQi-S, but only for the happy and sad go stimuli and for the fearful no-go stimulus conditions. Finally, with the TMMS, higher scores on the attention scale were related to higher FA rates when a sad face was the go stimulus. Multiple regressions only identified the managing branch as the predictor variable for the total FA rate. As we hypothesized, the results with the MSCEIT were thus more consistent; using the performance-based ability model, higher EI was found to be particularly beneficial to controlling incorrect responses. This result supports our previous suggestion that a slower response could be a better strategy for achieving the goal of the task. In other words, higher-EI participants were slower but more accurate, which matched the aim of their task.

Nonetheless, it is important to adequately understand this result, which led us to question of whether the lower-EI participants’ poorer performance on the FA index was due to poorer cognitive control ability or whether this could be a consequence of an additional mechanism involving a deficit in the perception of the stimuli. To this end, we examined the d-prime index, which showed a positive correlation with the managing branch of the MSCEIT when the go stimulus was an angry face and a positive correlation with the managing and perceiving branch of the MSCEIT with the total d-prime. In addition, multiple regressions revealed how the perceiving and managing branch of the MSCEIT predict the total d-prime, suggesting that higher-EI participants showed better discrimination under these conditions. Thus, the superior performance of these participants could be due to their greater ability to discriminate between the stimuli, along with an enhanced cognitive control capacity; the fact that for the remainder of the blocks, the FA scores were higher suggests better cognitive control ability—but not better discriminative ability—under these conditions. In addition, these analyses showed how the total d-prime score was more strongly predicted by the MSCEIT perceiving branch than the managing branch. This finding is unsurprising, given that the d-prime index is a measure of the ability to discriminate between stimuli. Thus, those participants with higher scores on the perceiving branch of the MSCEIT discriminate better than those with lower scores, a finding that also gives validity to the MSCEIT scale. In the case of the EQi-S, the lower FA rates for the higher stress-management participants in the fearful no-go condition may also be explained by their superior ability to discriminate the facial expressions, given the participants’ higher scores on the d-prime index under these conditions.

These results contribute towards confirming the validity of the performance-based ability model. Those individuals in our study who adequately managed their emotions presented consistently better cognitive control capabilities in an emotional context than those without such emotional management abilities. Our results are consistent with previous studies that have measured related cognitive control abilities with emotional stimuli. These studies also found that MSCEIT was negatively correlated with a Stroop task index (Martin and Thomas, 2011; Checa and Fernández-Berrocal, 2015). Further, Casey et al. (2011) demonstrated how pre-school individuals who showed difficulty in delaying gratifications, performed more poorly in a hot go/no-Go task than those who showed higher levels of delayed gratification (up to a period of 40 years). These findings support the hypothesis that the emotion-regulation branch within the Mayer and Salovey (1997) model of EI is likely to play a central role in determining personal and social success, since it entails awareness of the most effective strategies for creating adapted responses in novel emotional situations (Côté, 2014; Cabello and Fernández-Berrocal, 2015b; Peña-Sarrionandia et al., 2015).

Some researchers have also demonstrated that EI and cognitive control appear to be related to frontal brain regions. For instance, Krueger et al. (2009), using the MSCEIT, showed how “ability EI” depends on different regions of the prefrontal cortex (PFC). In particular, the ventromedial PFC appears to be related to the understanding and managing EI branches, while the dorsolateral PFC is related to the perception of emotions. In addition, Jausovec et al. (2001) demonstrated that low-EI individuals showed low activity in the left frontal cortex. Several studies have also related cognitive control to the frontal cortex (Miller, 2000; Ridderinkhof et al., 2004; Rushworth et al., 2004; Tang et al., 2016). Thus, cognitive control appears to sequentially activate various frontal regions, such as the dorsolateral PFC, the medial frontal cortex, and the orbitofrontal PFC (Tang et al., 2016).

When we focused on cool go/no-go tasks in our study, we only found differences between performance-based ability EI and the RT index. In this case, higher scores on the “perceiving” branch of the EI instrument were correlated with shorter RTs. The fact that the higher-EI participants displayed faster responses in this neutral task (in contrast with their slower responses during hot tasks) could be due to the relatively simple nature of the stimuli (red and green circles), which may not require the same depth of processing as the facial expressions that were used in the hot go/no-go tasks. For the FA and d-prime indices, as we hypothesized, no differences were found when the stimuli were neutral, thus suggesting better performance for higher-EI individuals only when the stimuli are emotionally laden, a finding that is consistent with the results reported by Gutiérrez-Cobo et al. (2016). This result, however, could be open to an alternative explanation. In particular, when analyzing the difficulty of the cool and hot tasks, we found the hot task to be significantly more challenging (total error percentage of 13.62%) than the cool task (total error percentage of 3.18%), which is also consistent with previous studies (Schulz et al., 2007). Given its relative lack of complexity, the cool go/no-go task could thus be insensitive to individual differences.

EI has been shown to have a beneficial effect during stressful situations (Slaski and Cartwright, 2003; Schneider et al., 2013; Extremera and Rey, 2015; Limonero et al., 2015; Peña-Sarrionandia et al., 2015). The hot go/no-go task could be regarded as analogous to a real-life stress situation, and in particularly stressful contexts, high EI would favor the liberation of working memory and attentional resources in order to adequately cope with the stressful situation, thus reducing (in this case) the incidence of FAs. Future researchers should analyze whether the differences between EI and cognitive control in hot and cool cognitive tasks are due to this emotional context or to the complexity of the tasks. In addition, it could be worthwhile to explore the effects of EI on working memory load capacity. Although cool and hot cognitive tasks are designed to measure the same cognitive processes (Schulz et al., 2007), the present study only found correlations in the RT index, which may be due to the fact that these tasks do not actually measure the same process or, again, because of the differences in complexity between the different tasks.

In addition, our results suggest that an improvement in the ability to manage emotions may enhance the capacity for cognitive control. Given the fact that a lack of cognitive control can have negative consequences for certain behaviors such as impulsiveness, drug abuse, or over-consumption (Aichert et al., 2012; Volkow et al., 2013; Holmes et al., 2016) this emotional management could help to control these undesirable behaviors, through the higher ability to control prepotent and unpleasant behaviors in critical situations. It is important to consider this clinical aspect due to the long-term relevance of the cognitive control ability (Casey et al., 2011).

It is also important to note that although the three instruments employed for measuring EI are designed to cover the EI construct, the correlations that we found between the MSCEIT and the TMMS and EQi-S were all very low (all *r* < 0.20), which was found to be the case for all of the scales. The strongest correlations were between the two self-report measures (from 0.25 to 0.43), which could possibly reflect the subjective nature of both tests; in particular, these correlations could have been inflated by common-method variance (Goldenberg et al., 2006; Webb et al., 2013; Cabello and Fernández-Berrocal, 2015a). Future researchers should pay attention to this important issue when choosing to employ the EI model in their studies.

A further issue concerns the results obtained for the hot go/no-go task. Statistical analyses showed that this task is suitable for measuring differences between emotions in the several indices employed. Overall, we discovered that fearful faces were those with higher RTs and FA, and lower d-prime score, in contrast to happy faces presenting the lower RT and FA, as well as the higher d-prime scores. It is important to mention that some of the effect sizes are rather small, such as the FA and d-prime interactions (0.02 and 0.03, respectively). However, these results are consistent with Tottenham et al. (2011) who found similar effects. It is possible that higher effect sizes make it easier to find stronger correlations between this task and the EI scales.

There are four important limitations related to the sample used in the present study. First, our results were obtained using an unrepresentative sample, given that it was composed of undergraduate students whose EI scores are susceptible to slight variations, and it is therefore possible that their performance is not representative of that of the general population. Second, our sample was predominantly composed of females, with only 25% of the participants being male. It is thus possible that gender differences could have had an impact on the EI scores (and therefore on the validity of our results and conclusions; Cabello et al., 2016). Third, we have not assessed if participants have suffered any neurological or psychiatric disorder and drug or alcohol abuse. Finally, interpreting the correlational analyses requires some caution due to multiple comparisons.

The go/no-go task is a well-known measure that has been employed in numerous studies. To the best of our knowledge, however, the current study is the first to examine the relationship between the go/no-go task and the EI construct. The present work has shown that higher-EI participants—measured primarily with the performance-based ability model instrument MSCEIT—demonstrated better cognitive control in hot go/no-go tasks in comparison with a cool version of the cognitive task. The results for the self-report measures of EI were less consistent across the conditions, although they did display some correlations with the cognitive control index, thus suggesting better performance for higher-EI individuals. Future research should focus on a more in-depth analysis of the relationship between the go/no-go task and the EI construct by using event-related potential measures that would provide more information about the mechanisms underlying the various cognitive control processes.

## Author Contributions

MJG-C: substantial contributions to the design of the work and interpretation, acquisition and analysis of data for the work; drafting the work critically for important intellectual content; final approval of the version to be published and agreement to be accountable for all aspects of the work in ensuring that questions related to the accuracy or integrity of any part of the work are appropriately investigated and resolved. RC: substantial contributions to the conception of the work and interpretation of data for the work; revising the work critically for important intellectual content; final approval of the version to be published and agreement to be accountable for all aspects of the work in ensuring that questions related to the accuracy or integrity of any part of the work are appropriately investigated and resolved. PF-B: substantial contributions to the conception of the work and interpretation and analyses of data for the work; revising the work critically for important intellectual content; final approval of the version to be published and agreement to be accountable for all aspects of the work in ensuring that questions related to the accuracy or integrity of any part of the work are appropriately investigated and resolved.

## Conflict of Interest Statement

The authors declare that the research was conducted in the absence of any commercial or financial relationships that could be construed as a potential conflict of interest.
